# Pesticides in Surface Drinking-Water Supplies of the Northern Great Plains

**DOI:** 10.1289/ehp.9435

**Published:** 2007-05-15

**Authors:** David B. Donald, Allan J. Cessna, Ed Sverko, Nancy E. Glozier

**Affiliations:** 1 Environment Canada, Regina, Saskatchewan, Canada; 2 Agriculture and Agri-Food Canada, Saskatoon, Saskatchewan, Canada; 3 National Laboratory for Environmental Testing, Environment Canada, Burlington, Ontario, Canada; 4 National Hydrology Research Centre, Environment Canada, Saskatoon, Saskatchewan, Canada

**Keywords:** drinking water, northern Great Plains, pesticides, reservoirs, water treatment

## Abstract

**Background:**

Human health anomalies have been associated with pesticide exposure for people living in rural landscapes in the northern Great Plains of North America.

**Objective:**

The objective of this study was to investigate the occurrence of 45 pesticides in drinking water from reservoirs in this area that received water primarily from snowmelt and rainfall runoff from agricultural crop lands.

**Methods:**

Water from 15 reservoirs was sampled frequently during the spring pesticide application period (early May to mid-August) and less frequently for the remainder of the year. Drinking water was sampled in early July. Sample extracts were analyzed for pesticide content using mass spectrometric detection.

**Results:**

We detected two insecticides and 27 herbicides in reservoir water. Consistent detection of a subset of 7 herbicides suggested that atmospheric deposition, either directly or in rain, was the principal pathway from fields to the reservoirs. However, the highest concentrations and number of herbicides in drinking water were associated with runoff from a localized 133-mm rainfall over 15 days toward the end of spring herbicide application. Water treatment removed from 14 to 86% of individual herbicides. Drinking water contained 3–15 herbicides (average, 6.4).

**Conclusions:**

We estimated the mean annual calculated concentration of herbicides in drinking water to be 75 ng/L (2,4-dichlorophenoxy)acetic acid, 31 ng/L (2-chloro-4-methylphenoxy)acetic acid, 24 ng/L clopyralid, 11 ng/L dichlorprop, 4 ng/L dicamba, 3 ng/L mecoprop, and 1 ng/L bro-moxynil. The maximum total concentration of herbicides in drinking water was 2,423 ng/L. For the seven herbicides with established drinking water guidelines, all concentrations of the individual chemicals were well below their respective guideline. However, guidelines have not been established for the majority of the herbicides found in drinking water or for mixtures of pesticides.

In agricultural landscapes, rural and municipal residents can be exposed to agricultural pesticides either directly during crop applications or indirectly in air, water, or food. In the northern Great Plains of the United States and Canada, pesticides have been detected in atmospheric samples, in surface and groundwaters, and in a variety of food products. Studies in the United States (Garry et al. 1996), Spain (García-Rodríguez et al. 1996), and New Zealand (Hanify et al. 1981) have shown that environmental exposure to agricultural chemicals is associated with increases in human health anomalies. These include reduced stamina, gross and fine eye–hand coordination, and cognitive abilities in children (Guillette et al. 1998); an increased incidence of human birth malformations (Garry et al. 1996; Hanify et al. 1981; Schreinemachers 2003); and cryptorchidism in male children (García-Rodríguez et al. 1996).

Pesticide exposure through potable water has become a concern. Using a statewide survey of 856 Iowa municipal drinking water supplies, Munger et al. (1997) compared the rate of intrauterine growth retardation in births by women whose drinking water contained higher levels of herbicides [atrazine, cyanazine, metolachlor, and (2,4-dichloro-phenoxy)acetic acid (2,4-D)] with that in births by women using other sources of drinking water. The authors concluded that atrazine, metolachlor, and cyanazine were each significant predictors of intrauterine growth retardation and that areas with drinking water containing higher herbicide concentrations had higher rates of intrauterine growth retardation than nearby communities with other sources of drinking water.

In the northern Great Plains of Canada and the United States, drinking water sources include groundwater and large rivers. However, because of inadequate volume or unsuitability of groundwater because of high mineral content, residents of some smaller communities derive their drinking water from small reservoirs with drainage areas imbedded in agricultural landscapes. In a 3-year study, Cessna and Elliott (2004) monitored prairie farm dugouts (constructed ponds) in Saskatchewan for several herbicides used extensively in crop production on the Canadian prairies. Two of these small reservoirs were used by the farm families for drinking water and household water. Herbicides detected in these dugouts were those expected from an agricultural landscape dominated by cereal and oilseed production and included 2,4-D, diclofop, bromoxynil, (2-chloro-4-methylphenoxy)acetic acid (MCPA), triallate, dichlorprop, dicamba, clopyralid, and tri-fluralin. Consequently, we hypothesized that rural populations obtaining drinking water from catchments that are predominantly crop lands may be exposed to relatively high concentrations of pesticides in drinking water. In the present study, we assessed the potential for occurrence of pesticides in drinking water of residents of 15 rural communities situated in the northern Great Plains in Canada (Figure 1).

## Materials and Methods

### Study sites

The 15 communities, associated drinking water reservoirs, and water treatment plants were in Manitoba, Saskatchewan, and Alberta and had populations ranging from 95 to 10,959 (Table 1). We intentionally selected communities where the source of drinking water in reservoirs was primarily from snowmelt runoff from crop lands, although occasionally rainfall runoff can also be a significant source of water to these reservoirs. In this region, evaporation exceeds precipitation, and rainfall runoff is a relatively rare event. None of the reservoirs was equipped with a meteorologic station, and rainfall data used to assess the occurrence of surface runoff were from the nearest Environment Canada weather station.

Sources of pesticides to the reservoirs included snowmelt and rainfall runoff. Pesticides present in the atmosphere due to application drift, postapplication vapor loss, and wind erosion of soil also entered the reservoirs through both wet (precipitation) and dry (particulate) deposition. Natural vegetation, which provided some protection to the reservoirs through mitigation of surface runoff and atmospheric deposition, covered ≤ 10% of the drainage area for each of the selected reservoirs. Because of poor groundwater quality (Corkel et al. 2004), these reservoirs are generally sited to avoid hydrologic recharge from ground-water. Finally, there was no flood irrigation in any of the catchments, so irrigation runoff would not have been a contributing factor.

#### Reservoir and water characteristics

The storage capacity of the reservoirs varied from 41.6 to 38,800 decameter^3^ with maximum depths that ranged from 4 to 15.8 m (Table 1). Surface areas varied from 0.8 to 1,090 ha, and drainage areas ranged from 3.0 to 15,500 km^2^. Water temperature profiles from individual reservoirs indicated that, with few exceptions, the water in the reservoirs remained weakly thermally stratified during summer.

In midsummer (July 2003), the water in the reservoirs varied in dissolved chemical composition for a wide range of parameters. In general, the order of decreasing cation concentrations was as follows: calcium (19–111 mg/L) > sodium (11.9–332 mg/L) > magnesium (8.2–82.1 mg/L) > potassium (3.5–19.5). For anion concentrations, the order was bicarbonate (99–374 mg/L) > sulfate (36–674 mg/L) > chloride (4.9–41.8 mg/L) > fluoride (0.1–0.32 mg/L) > nitrate (< 0.01–0.33 mg/L). Concentrations of total dissolved solids were 140–891 mg/L; dissolved organic carbon, 6.8–20.4 mg/L; total phosphorus, 0.02–1.05 mg/L; and total nitrogen, 0.43–1.57 mg/L. Total alkalinity [as calcium carbonate (CaCO_3_)] ranged from 90.6 to 336 mg/L, total hardness from 97 to 515 mg/L (as CaCO_3_), and ammonia concentrations from 0.016 to 0.308 mg/L. The reservoir waters were slightly alkaline (pH 8.08–9.12). In general, the midsummer concentrations of these water quality parameters in the reservoirs were less than Canadian drinking water guidelines [Canadian Council of Ministries of the Environment (CCME) 1999], although there were exceptions at some sites for total dissolved solids and sulfate.

#### Water treatment

Water treatment in the communities was generally similar (Table 2). Treatment included pretreatment aeration and copper sulfate application at some of the smaller reservoirs, alum and/or potassium permanganate addition at the treatment plant (to induce precipitation and settling of the flocculent), sand filtration, and finally chlorination prior to distribution to the community. Twelve communities had some form of activated carbon treatment; one community also used membrane filtration in their treatment process.

#### Water sampling

Reservoir water samples for pesticide analyses were collected near the center of each reservoir at a depth of 2 m. In 2003, we collected reservoir water samples every 2 weeks from early May through mid-August to coincide with spring application of herbicides (May to early July) and organophosphorus insecticides (mid- to late July). We also collected water samples once before ice formation (October 2003), through the ice in midwinter (January 2004), and after spring snowmelt runoff (April 2004 and 2005). We collected simultaneous reservoir and treated drinking water samples in early July 2004 and 2005. Drinking water samples were collected after water treatment at the beginning of each distribution system where water was first accessed for drinking by the community. Pesticide concentrations in these paired samples were used in a general assessment of pesticide reduction by the water treatment plant associated with each reservoir.

We collected water samples for pesticide analyses in four separate 1-L amber glass bottles, one each for analysis of the acid, neutral, and sulfonylurea herbicides, and one for organophosphorus insecticides. The acid herbicide samples were preserved with 2 mL of concentrated, pesticide-grade sulfuric acid, and all samples were maintained at 4°C in the dark until analysis.

Although intense rainfall events are rare in this region, the Pembina River catchment (7,500 km^2^), which either incorporates or is near the four reservoirs in southern Manitoba, was subjected to an unusually high average rainfall of 133.3 mm during the 15 days before our scheduled July 2005 water sample collection at the end of the herbicide application period. During the same period of the previous year of the study, only 22.3 mm of rain (17% of the corresponding 2005 rainfall) occurred before reservoir and drinking water samples were collected. Normal total precipitation for this 15-day period is 45 mm. The 30-day rainfall in the Pembina River catchment was 61 mm and 201 mm before early July water sample collections for 2004 and 2005, respectively. Mean daily discharge for the Pembina River from 21 June to 5 July was 14.8 m^3^/sec in 2004 and 47.9 m^3^/sec in 2005, indicating that the June–July precipitation in 2005 generated significant surface runoff. Therefore, in a separate analysis, we compared concentrations and number of herbicides in drinking water samples for the same four reservoirs with these two precipitation regimes. None of the other reservoirs was subjected to this magnitude of difference in rainfall for June 2004 and 2005. Rainfall data for the Pembina basin were obtained from meteorologic stations with the following latitudes and longitudes: 49°10′N, 98°04′W; 49°39′N, 100°15′W; 49°15′N, 98°31′W; and 49°10′N, 99°39′W.

### Pesticide residue analysis

The 45 pesticides and degradation products assessed during the study included 17 acidic herbicides [2,4-D, MCPA, (2-chloro-4-methylphenoxy)butyric acid (MCPB), 4-(2,4-dichlorphenoxy) butanoic acid (2,4-DB), (2,4,5-trichlorophenoxy)acetic acid (2,4,5-T), 2,3,6-trichloro-benzoic acid (2,3,6-TBA), benzoylprop, bromoxynil, clopyralid, dicamba, dichlorprop, diclofop, fenoprop, imazamethabenz A and B, imazethapyr, mecoprop, and picloram], 8 neutral herbicides (atrazine, butylate, diallate, metolachlor, metribuzin, simazine, triallate, and trifluralin), 5 sulfonylurea herbicides (ethametsulfuron-methyl, metsulfuron-methyl, thifensulfuron-methyl, tribenuron-methyl, and sulfosulfuron; hereafter, “-methyl” has been dropped from the formal names of the sulfonylurea herbicides), 2 herbicide degradation products (desethylatrazine and desethylsimazine), and 13 organophosphorus insecticides (azinphos, chlorpyriphos, diazinon, dibrom, dimethoate, disulfoton, ethion, fonofos, malathion, parathion, phorate, phosmet, and terbufos).

#### Acid herbicides, neutral herbicides, and organophosphorus insecticides

We analyzed the acid and neutral herbicide and organo-phosphorus insecticide water samples at Environment Canada (National Laboratory for Environmental Testing, Burlington, Ontario, Canada). To assess recovery of the pesticides from the reservoir and drinking water samples, we added the surrogate compounds [2,3-dichlorophenoxyacetic acid, deuterium-labeled d14-trifluralin, and d10-malathion in acetone (100 μL)], to the acid herbicide, neutral herbicide, and organophosphorus insecticide water samples, respectively, before sample extraction such that the corresponding concentrations were 20, 46, and 29 μg/L.

#### Sample extraction

We extracted the neutral herbicide and organophosphorus insecticide water samples (1 L) with dichloromethane. The acid herbicide water samples (1 L) were first acidified to pH 2 with 50% sulfuric acid and then extracted with dichloromethane.

The neutral herbicide and organophosphorus insecticide extracts were concentrated (~ 5 mL) using Kuderna-Danish evaporation and quantitatively transferred to a test tube; *iso*-octane (2 mL) was added, and then the sample was evaporated to approximately 1.0 mL using a gentle stream of nitrogen gas. The organophosphorus insecticide extracts were transferred onto silica gel (deactivated with 10% water) cleanup columns and eluted with 10% acetone in hexane. The neutral herbicide extracts were transferred onto Florisil (deactivated with 10% water) cleanup columns and eluted with 2% methanol in dichloromethane. Eluates from both cleanup columns were concentrated to 1 mL volume before gas chromatographic analysis.

The acid herbicide extracts were similarly evaporated and transferred to a test tube and evaporated to dryness using a gentle stream of nitrogen gas; the extract residue was then dissolved in acetone (4 mL). Pentafluorobenzyl bromide (5% wt/vol in 200 μL acetone), together with potassium carbonate (30% wt/vol in 30 μL deionized water), was added and the mixture heated at 60°C for 3 hr to form the pentafluorobenzyl esters. *Iso*-octane (2 mL) was added and the reaction mixture evaporated to approximately 1.0 mL using a gentle stream of nitrogen gas. The sample extracts were transferred to silica gel (deactivated with 5% water) cleanup columns topped with anhydrous sodium sulfate (0.5 cm), the columns eluted with 5% methanol in toluene, and the eluate concentrated to a 1-mL volume before gas chromatographic analysis.

#### Gas chromatography–mass spectrometric analysis

We analyzed the organophosphorus insecticide and derivatized acid herbicide water sample extracts by gas chromatography-negative-ion-chemical-ionization mass spectrometry. We used a model 6890 gas chromatograph interfaced with a model 5973 mass selective detector operated in selected ion monitoring mode (Agilent Technologies, Wilmington, DE, USA) with a DB-5 column (30 m × 0.25 mm i.d.; 0.25 μm film thickness; Agilent Technologies, Palo Alto, CA, USA) with methane as the moderating gas. The neutral herbicide extracts were analyzed with the same instrument in the electron ionization mode. All pesticide concentrations were quantified against 5-point calibration curves from the targeted analyte mixture (Sigma-Aldrich Canada Ltd., Oakville, Ontario, Canada), and the two most abundant isotopic ions of each parent ion were monitored.

Quality assurance/quality control measures included a laboratory blank sample (type I water) and two fortified laboratory blank samples with every 12 reservoir or drinking water samples. No compounds were detected in the laboratory blank samples. Recoveries for the laboratory blank samples fortified with the 17 acidic herbicides, 8 neutral herbicides, 2 herbicide degradation products, and 13 organophosphorus insecticides (concentrations of 10–150 ng/L) varied from 71 to 124% (*n* = 36). The laboratory surrogate recoveries for both quality assurance/ quality control and field samples ranged from 72 to 115%.

#### Sulfonylurea herbicides

We analyzed the sulfonylurea herbicide water samples at the National Hydrology Research Centre in Saskatoon, Saskatchewan.

#### Sample extraction

We passed the reservoir and drinking water samples (500 mL) through solid-phase extraction cartridges under a vacuum of 400 mm of Hg (~ 10 mL/min). The cartridges (Oasis HLB extraction cartridges; Waters Corporation, Milford, MA, USA) were conditioned sequentially with methanol (10 mL) and then deionized water (10 mL). After sample loading, the cartridges were washed with deionized water (10 mL) and then dried for 1 hr under vacuum. After drying, the cartridge was eluted with methanol (10 mL) and the eluate evaporated to dryness using a stream of dry nitrogen gas (water bath at 50°C). The residue was dissolved in deionized water (1 mL) and transferred to a 2-mL HPLC (high-performance liquid chromatography) vial. [Because sulfonylurea herbicides may hydrolyze in water, the methanol evaporation and subsequent dissolving of the extract residue in deionized water should be carried out just prior to analysis by liquid chromatography-tandem mass spectrometry (LC-MS-MS).]

#### Liquid chromatography–mass spectrometric analysis

We used a Waters 2695 Alliance HPLC system with a Waters Xterra Mass C_18_ (100 mm × 2.1 mm i.d., 3.5 μm diameter particle size) analytical column (both from Waters Limited, Mississauga, Ontario, Canada) which was maintained at 30°C. Mobile phase consisted of solvent A (90:10 water:acetonitrile) and solvent B (90:10 acetonitrile:water). Both solvents contained 0.1% formic acid and 2 mM ammonium acetate. Isocratic elution of the column with 70% solvent A and 30% solvent B at a flow rate of 200 μL/min resulted in retention times of 4.81, 5.57, 8.05, 12.41, and 14.22 min for thifensulfuron, metsulfuron, ethametsulfuron, sulfosulfuron, and tribe-nuron, respectively. All injection volumes were 20 μL.

We quantitated the sulfonylurea herbicides and confirmed their presence using the Waters Micromass Quattro Ultima triple quadrupole mass spectrometer (Waters Limited) equipped with an electrospray ionization interface set to positive ion mode. Ionization and MS-MS conditions were optimized by infusing a 0.5-mg/L solution of each sulfonylurea herbicide into the ion source in a 50:50 acetonitrile:water solution with a syringe pump. The (M+H)^+^ ion for each analyte was selected for fragmentation using the first quadrupole; the second quadrupole, into which argon gas was introduced, functioned as a collision cell; and the third quadrupole was used to monitor the resulting major fragment ion.

Suitable multiple reaction monitoring transitions were chosen from the product ion scans and were as follows: thifensulfuron, 388.3 to 167.2 atomic mass units (amu); metsulfuron, 382.3 to 167.2 amu; ethamet-sulfuron, 411.3 to 196.3 amu; sulfosulfuron, 471.3 to 261.2 amu; and tribenuron, 396.3 to 155.2 amu. Instrument operating conditions have been described previously (Cessna et al. 2006).

Recovery of the five sulfonylurea herbicides was determined from both deionized and reservoir water. Water samples (500 mL) were fortified with 5 or 50 ng of each sulfonylurea herbicide dissolved in 100 μL acetonitrile resulting in concentrations of 10 and 100 ng/L, respectively. Mean recoveries of thifensulfuron, metsulfuron, ethamet-sulfuron, sulfosulfuron, and tribenuron from deionized water ranged from 73 to 84% at 100 ng/L (*n* = 13) and from 81 to 123% at 10 ng/L (*n* = 13). Corresponding mean recoveries from reservoir water were 105–115% (*n* = 10) and 82–108% (*n* = 10), respectively.

The high recoveries from the fortified reservoir waters may indicate ionization enhancement in the source of the mass spectrometer due to the relatively high content of dissolved organic matter in these waters. Herbicide recoveries indicated that the solid-phase extraction method was effective for both deionized and reservoir waters; when coupled with electrospray ionization MS-MS quantification and confirmation, this method provided reliable recoveries down to 10 ng/L. The instrumental limit of quantification was approximately 20 pg for each herbicide and, assuming 100% herbicide extraction recovery from a 500-mL water sample, was equivalent to a method limit of quantification of 2 ng/L.

### Statistical analyses

We performed statistical analyses using Systat, Version 11 (Systat Software Inc., Point Richmond, CA, USA) for *t*-tests and Primer, Version 5.2.9 (Primer-E Ltd., Plymouth, UK) for principal component analysis (PCA). In cases where pesticides were not detected, we used values equal to one-half the limit of quantification for statistical calculation and graphic presentation (Gilbert 1987). For the parametric statistical analyses, data were examined for heteroscedasticity (unequal variances) or departures from normality, and when found, appropriate transformations were applied before analyses. To examine patterns in herbicide concentration across the northern Great Plains, we performed a multivariate, PCA. PCAs essentially combine the results of all parameters measured into a two-dimensional space where sample similarities can be highlighted, thus allowing patterns between sets of samples to be recognized. Parameters that are correlated with any apparent patterns can also be identified. We selected *p* < 0.05 for statistical significance; results are reported as mean ± 1 SE and actual *p*-values, unless otherwise noted.

## Results

### Pesticides in reservoir water

Of the 45 pesticides and degradation products monitored during the study, we detected 2 insecticides, 27 herbicides, and 2 degradation products in water collected from 15 reservoirs (*n* = 206; Table 3). These included 16 acid herbicides, 6 neutral herbicides, 5 sulfonylurea herbicides, 2 herbicide degradation products, and 2 organophosphorus insecticides. Of the 31 analytes detected, three (2,4-D, clopyralid, and dicamba) were present in the reservoirs at concentrations > 1,000 ng/L. Six additional herbicides (dichlorprop, MCPA, metribuzin, picloram, imazamethabenz A, and bromoxynil) were detected at concentrations > 100 ng/L, with the remainder (22) at concentrations < 100 ng/L. We did not detect 2,4-DB, dial-late (*cis*- and *trans*-isomers), metolachlor, or the majority of organophosphorus insecticides (azinophos, diazinon, dibrom, disulfoton, ethion, fonofos, malathion, parathion, phorate, phosmet, or terbufos). With few exceptions, pesticides that were not detected are not normally used in the study area.

**Figure 2:**
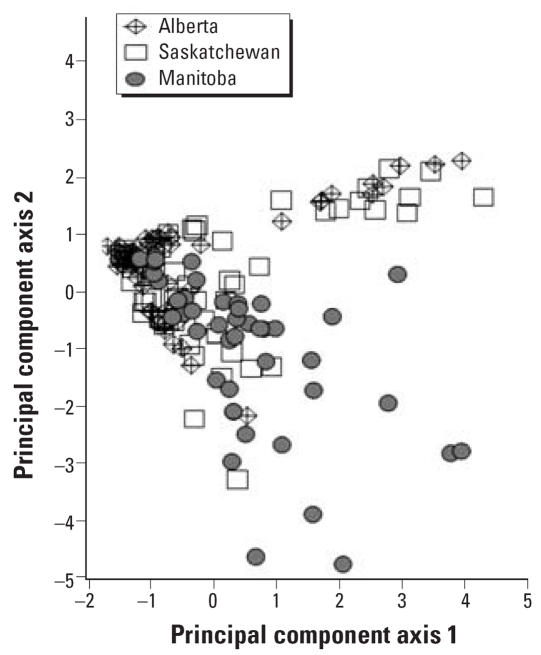
PCA of concentrations of 2,4-D, MPCA, clopyralid, dichlorprop, dicamba, mecoprop, and bromoxynil in 163 water samples collected from 15 reservoirs from May 2003 to April 2004 (44 samples from Manitoba, 66 samples from Saskatchewan, and 53 samples from Alberta).

Seven herbicides were consistently present in water samples from the 15 drinking water reservoirs (2,4-D, MCPA, clopyralid, diclor-prop, dicamba, mecoprop, bromoxynil; Table 4). Although mean concentrations for these individual herbicides varied across the reservoirs by as much as 20- to 50-fold, a principal component analysis suggested no distinct geographic pattern of herbicide concentrations. Samples from all three Canadian prairie provinces showed extensive overlap in the two-dimensional pattern (Figure 2), indicating that concentrations in this mixture of seven herbicides are not related to geographic location. Mean total herbicide concentration was not correlated with reservoir storage capacity (*r* = 0.17; *n* = 15). Clopyralid, 2,4-D, and MCPA were detected in essentially all of the reservoir samples taken throughout the sampling period, regardless of time of year (Table 3). Dicamba, diclorprop, and mecoprop were detected in > 75% of the samples and bromoxynil in 54%. The overall mean concentrations of these seven herbicides in the reservoirs from May 2003 to April 2004 were, in decreasing order: 123 ng/L 2,4-D, 57 ng/L MCPA, 28 ng/L clopyralid, 16 ng/L dichlorprop, 6.6 ng/L dicamba, 4.4 ng/L mecoprop, and 2.4 ng/L bromoxynil (Table 5; *n* = 163 samples).

The total number of herbicides detected during the study was similar for all three provinces (22 in Manitoba and Saskatchewan and 24 in Alberta). However, for herbicides other than the seven discussed above, some regional differences were evident (Table 4). Differences included a higher frequency of detection of atrazine and sulfonylurea herbicides in reservoirs in southern Manitoba, whereas picloram was detected only in reservoirs in Alberta.

**Figure 3:**
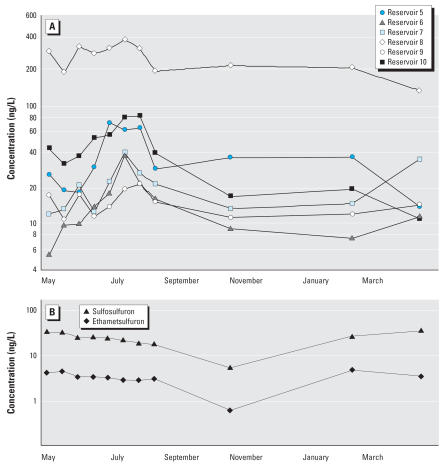
Seasonal concentration from May 2003 to April 2004 of MCPA in six reservoirs in Saskatchewan (*A*) and sulfosulfuron and ethametsulfuron in reservoir 4 in Manitoba (*B*).

For those herbicides consistently detected in July, we tested if concentrations were significantly greater in July samples than in early spring samples (April/May) with a one-way, paired two-sample *t*-test (*p* < 0.05, data were log-transformed to equalize variances). For six herbicides (bromoxynil, MCPA, 2,4-D, diclorprop, dicamba, and clopyralid), concentrations were significantly greater in July samples than in early spring (Table 6, Figure 3A, MCPA only). Most of these herbicides exhibited a 2- to 4-fold increase except for bromoxynil, which showed a 20-fold increase in concentration from April/May to July. For the other herbicides tested (mecoprop, tribenuron, ethametsulfuron, and imazamethabenz A and B), concentrations were higher in July samples but did not differ significantly from those in the April/May samples. Finally, because there was an extreme rain event in Manitoba in the summer of 2005, we performed the paired *t*-tests with and without the July 2005 samples; the significance of the analyses was unaffected by inclusion or exclusion of these samples in the analysis. Other herbicides, such as the sulfonylurea herbicides, generally had similar concentrations throughout the year (Figure 3B).

### Effect of water treatment

Drinking water contained an average of 6.4 herbicides (*n* = 28 samples), with the number ranging from 3 to 15 depending on the location. We detected 21 herbicides in the 28 drinking water samples. The reservoir and drinking water samples collected simultaneously in early July indicated that water treatment at these communities reduced herbicide concentrations by an average of 14–86%, depending on the herbicide (Table 5). However, percent reduction (based on individual herbicides detected in both reservoir and drinking water samples) was highly variable from one treatment facility to another, and often between years at the same facility. Water treatment generally reduced bro-moxynil, dichlorprop, dicamba, mecoprop, imazethabenz, and atrazine concentrations to nondetectable levels in the drinking water when reservoir concentrations were < 20 ng/L.

The highly variable reduction in herbicide concentrations (Table 5) showed no obvious relation to differences in treatment procedures. For example, for six water treatment facilities, reduction of 2,4-D concentrations differed by at least 30% between 2004 and 2005. However, our data showed little difference in 2,4-D reduction between the three largest facilities with the more sophisticated water treatment procedures and the three smallest facilities (mean 2,4-D reduction of 37% and 38%, respectively). In another treatment comparison, MCPA reduction was not significantly different among those facilities that used potassium permanganate (42.3%, *n* = 12) and those that did not [48.0%, *n* = 14; two sample *t*-test with arcsine (square root) transformation, *p* = 0.62]. However, the community with the most sophisticated treatment technology (membrane filtration) had the highest average removal rate for dichlorprop (47%), chlopyralid (59%), and MCPA (> 95%) (mean for two samples), but not for 2,4-D or dicamba.

### Herbicides in drinking water

We calculated mean annual concentration of herbicides in drinking water from the average concentrations of herbicides in reservoirs (mean of means calculated from data in Table 4) times the mean percent of these same chemicals remaining after water treatment (Table 5). To calculate these estimates, we assumed that the average herbicide concentrations in reservoirs (determined for May, June, July, August, October, February, and April data) and mean percent reduction due to water treatment in July would be applicable annually. For the seven herbicides detected frequently across the northern Great Plains, mean annual concentrations in drinking water were 75 ng/L 2,4-D, 31 ng/L MCPA, 24 ng/L clopyralid, 11 ng/L dichlorprop, 4 ng/L dicamba, 3 ng/L mecoprop, and 1 ng/L bromoxynil. Our data show that, from time-to-time, residents in some communities were exposed to relatively high concentrations of a few of these chemicals in drinking water for short periods. For example, the mean annual concentration of 2,4-D could occasionally be as high as 364 ng/L at one of the 15 communities, and maximum concentrations of several pesticides in drinking water samples could be > 100 ng/L (Table 5).

In 2005, following unusually high rainfall, we detected record concentrations (for this study) in Manitoba reservoirs for 2,4-D (1,850 ng/L), clopyralid (1,050 ng/L), bromoxynil (384 ng/L), imazamethabenz A and B (288 ng/L), ethametsulfuron (80 ng/L), and tribenuron (30 ng/L). The region was subjected to total average rainfall of 133.3 mm in the 15 days before sample collection. Total herbicide concentrations detected in the four reservoirs were higher than corresponding total concentrations in 2004 by factors of 2.1 to 10.6 (Table 7). The maximum number (15) and maximum total concentration (2,423 ng/L) of herbicides in drinking water also occurred after the high rainfall and runoff in southern Manitoba (Table 7). In the four communities in 2005, total herbicide concentrations in the drinking water were higher than corresponding total concentrations in 2004 by factors of 1.1 to 8.3.

## Discussion

### Water treatment

We detected 2 insecticides, 27 herbicides, and 2 degradation products in reservoirs used as sources for drinking water by 15 communities in the northern Great Plains (Table 3). The insecticides were detected infrequently and at concentrations < 20 ng/L. Up to 15 herbicides were detected in single reservoir water samples. All of the communities had a water treatment facility and, on average, these reduced herbicide concentrations in the drinking water by 14–86% of those in the reservoir water (Table 5), depending on the herbicide, its concentration in the reservoir water, and, most likely, other factors. After treatment, however, 3–15 herbicides remained in potable water supplies at a combined concentration of < 2,500 ng/L.

Our results indicate that herbicide reduction at water treatment facilities was highly variable from one site to another and often from year-to-year. Furthermore, the results suggest that there were no obvious differences in herbicide reduction for different water treatment procedures. However, the single facility with membrane filtration had the highest average percent reduction for three of the five herbicides detected in drinking water at that facility. However, our study design provided only general estimates of pesticide reduction at specific water treatment facilities. To achieve greater precision, a larger number of samples would be required to improve statistical confidence, water samples would have to be collected exactly at the water intake of each facility (rather than midreservoir at a 2-m depth), and the water from that point tracked to the point of entry to the water distribution system.

### Pesticide mixtures

Drinking water guidelines have been established by Health Canada (CCME 1999) and other agencies for only seven of the herbicides commonly detected in drinking water. Individual herbicide concentrations in drinking water were usually one to three orders of magnitude lower than established guidelines. Even the total concentration of all herbicides in drinking water following excessive rainfall in Manitoba (Table 7) did not exceed the guideline for any individual herbicide (Table 5). These guidelines were set to protect humans from adverse health effects when continuously exposed over their lifetime to these herbicides in drinking water. However, drinking water guidelines have not been established for a much more complex issue—exposure to mixtures of pesticides. Monitoring programs throughout North America and Europe, together with the results of this study, have demonstrated the widespread presence of pesticide mixtures in surface waters. Thus, it is important to establish if the toxicity of a mixture of pesticides is different from the sum of the toxicities of the single compounds, or if two or more pesticides simultaneously present in drinking water have synergistic effects.

The toxicity of mixtures of pesticides in waters is now receiving greater attention in the literature. Cassee et al. (1998) provided a detailed discussion of toxicologic interactions between chemicals in mixtures, and Chèvre et al. (2006) presented a method of defining a risk quotient for mixtures of herbicides with similar modes of action. Toxicity of pesticide mixtures is also being assessed. Using enclosures in a prairie wetland, Forsyth et al. (1997) demonstrated a greater than additive (synergistic) effect when the submersed macrophytes *Potamogeton pectinatus* and *Myriophyllum sibiricum* were exposed to a mixture of 2,4-D and picloram. Porter et al. (1999) measured aggressive behavior, thyroxine hormone levels, and ability to make antibodies against a foreign protein in mice treated with atrazine, aldicarb, and nitrate (and their mixtures) at maximum concentrations typically detected in ground-water. Their results suggested that some mixtures, especially nitrate plus either pesticide, had effects not detected from exposure to the individual chemicals. Thus, when assessing environmental exposure involving mixtures of pesticides, single chemical evaluations of toxicity (e.g., Gandhi et al. 2000; Muir et al. 1991), although they provide useful information, generally have little practical value when assessing normal environmental exposure involving mixtures of pesticides.

In the context of mixtures, it is noteworthy that the 17 herbicides detected in the drinking water samples in the present study represent seven very different chemical classes: phenoxyalkanoic acids (2,4-D, mecoprop, MCPA, dichlorprop), sulfonylureas (ethametsulfuron, tribenuron, sulfosulfuron), pyridinecarboxylic acids (clopyralid, picloram), triazines (atrazine), hydroxybenzonitriles (bromoxynil), benzoic acids (dicamba, 2,3,6-TBA), and imidazolinones (imazamethabenz, imazethapyr). Thus, when mixtures of pesticides in drinking water include different chemical classes and, potentially, different modes of action, unexpected toxic effects may result.

The herbicides 2,4-D, MCPA, clopyralid, dichlorprop, dicamba, mecoprop, and bromoxynil are widely distributed in drinking water reservoirs in the northern plains. This consistent pattern suggests that these chemicals should be evaluated as a single “toxic substance” when assessed from the perspectives of human health and environmental effects. Based on the present study, the approximate ratio of these individual chemicals (relative to bromoxynil) in this toxic substance in reservoir water would be as follows: 2,4-D, 51; MCPA, 24; clopyralid, 12; dichlorprop, 7; dicamba, 3; mecoprop, 2; and bromoxynil, 1 (calculated from data in Table 5). Depending on the percent reduction achieved by water treatment facilities for the various herbicides, the relative ratios of chemicals in this toxic substance could change for drinking water. In the present study, the ratios in the drinking water were as follows: 2,4-D, 75; MCPA, 31; clopyralid, 24; dichlorprop, 11; dicamba, 4; mecoprop, 3; and bromoxynil, 1 (Table 5). The increase in the ratios in drinking water occurred because bromoxynil underwent the greatest reduction during water treatment. Because all of these herbicides have been used in the prairie region for at least the previous two decades (Pesticide Manual 2006), this current pattern may approximate that of past years.

### Source of pesticides to the reservoirs

Spring snowmelt runoff and atmospheric deposition are two potential transport routes for pesticides from fields to reservoirs. In 2003, after weeks of insignificant rainfall typical of the prairie region, some herbicides reached peak concentrations in the reservoirs in early July rather than after spring snowmelt runoff (Figure 3A). This pattern suggests that the atmospheric pathway, most likely involving both long-range transport and application drift (short-range transport; Grover et al. 1997), was dominant for these herbicides. Atmospheric transport appeared to be an important pathway to reservoirs for bromoxynil, MCPA, diclorprop, dicamba, 2,4-D, and clopyralid because these herbicides typically reached peak concentrations in early July in the absence of runoff. Long-range transport would include deposition of pesticides to the reservoirs in rain, on soil particles, and from direct transfer of pesticide from the atmosphere to the reservoir at the air–water interface. Relatively high concentrations of pesticides have been detected in the atmosphere (Grover et al. 1976; Rawn et al. 1999a; Waite et al. 2002), in rain (Hill et al. 2002; Rawn et al. 1999b; Strachan 1988), and on wind-eroded soil particles in the northern plains (Larney et al. 1999). In this region, mass balance calculations indicate that atmospheric deposition alone can account for the levels of herbicides detected in shallow aquatic habitats in the northern plains (Donald et al. 2001). Moreover, the relatively homogenous distribution of several herbicides evident in the reservoirs (Figure 2) is best explained by atmospheric transport and deposition to surface waters throughout the northern plains landscape.

Although atmospheric processes were most likely the principal mechanisms for movement of herbicides from fields to reservoirs, snowmelt and occasionally rainfall runoff probably contributed to the total loading of pesticides into the reservoirs (Muir and Grift 1987; Nicholaichuk and Grover 1983; Rawn et al. 1999c; Waite et al. 1992). Snowmelt runoff was probably an important source of herbicides, such as ethametsulfuron and imazamethabenz, to the reservoirs. After herbicide application to crops, major rainfall runoff from agricultural landscapes (Hunter et al. 2002) can transport relatively high concentrations of a variety of insecticides and herbicides to reservoirs and wetlands (Table 7) (Donald et al. 1999, 2005; Wauchope 1978). During the present study, the highest recorded concentrations of six herbicides in reservoir water followed 133 mm of rain in 15 days.

Management practices could be implemented to reduce concentrations of pesticides in small prairie reservoirs. This would require the cooperation of the landowners who farm the catchments surrounding reservoirs and could include organic farming, establishment of buffer strips of natural vegetation along field margins, and development of wildlife habitat along reservoir margins. Practices to reduce deposition of application drift to reservoirs might include decreased aerial application of pesticides near drinking water reservoirs, spraying when wind speeds are optimal, and use of precision applicators. Also, concentrations in runoff to reservoirs could be reduced through use of pesticides with lower water solubility. However, none of the above procedures would completely eliminate pesticides from drinking water reservoirs because long-range atmospheric transport and deposition from beyond reservoir catchments maintain detectable levels of a variety of herbicides in all surface waters in the northern plains.

## Conclusions

We detected a variety of pesticides at nanogram-per-liter levels in reservoirs that supply drinking water to small communities situated in the northern Great Plains. Water treatment in these communities reduced pesticide concentrations, but depending on the location, 3–15 herbicides remained in drinking water. Total concentrations of all pesticides generally were well below guidelines for individual pesticides; however, guidelines have been established for only 7 of the herbicides commonly detected in reservoir water, and no guidelines have been established for pesticide mixtures. Management practices could be implemented within drainage areas to lower the pesticide levels in small reservoirs and thereby improve the aesthetic quality and the safety of the water.

## Figures and Tables

**Figure 1:**
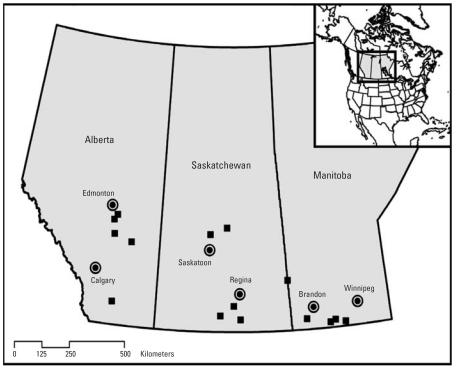
Study area showing the locations of drinking water reservoirs in Manitoba, Saskatchewan, and Alberta, Canada.

**Table 1:** Location of the 15 reservoirs, their morphology, and population of associated communities.

Reservoir	Site latitude N	Site longitude W	Surface area (ha)	Maximum depth (m)	Mean depth (m)	Storage capacity (dam^3^)^a^	Drainage area (km^2^)	2001 Pop.
Manitoba								
1	49°10′44″	98°08′09″	29.5	15.0	12.9	3,800	130	6,142
2	49°17′21″	98°37′40″	38.6	11.3	3.7	1,419	60.0	775
3	49°12′28″	98°56′54″	24.1	6.7	2.3	550	153	676
4	49°24′21″	100°00′47″	0.8	6.6	5.1	41.6	7.8	725
Saskatchewan								
5	50°54′58″	101°30′44″	0.8	6.7	5.3	44.7	5.1	95
6	49°27′48″	104°34′44″	11.7	6.6	2.5	290	25.6	105
7	49°59′10″	105°00′25″	227	6.4	3.3	7,413	64.7	412
8	52°58′46″	105°28′05″	6.5	5.2	5.1	330	77.3	957
9	49°36′36″	105°51′48″	67.0	15.8	7.8	5,215	39.2	2,483
10	52°47′73″	106°35′65″	1.2	5.5	5.5	63.2	3.0	236
Alberta								
11	52°13′27″	111°53′54″	20.7	6.1	2.5	518	84.0	970
12	49°52′51″	112°46′48″	40.0	4.0	2.0	740	15,500	1,669
13	53°12′53″	113°02′22″	4.0	4.0	4.0	225	4.0	352
14	52°28′18″	113°05′29″	8.0	4.0	4.0	475	620	487
15	53°00′26″	113°13′32″	1,090	5.5	3.5	38,800	125	10,959

Pop., population.

a Storage capacity (in cubic decameters) of each reservoir is design capacity and does not represent the volume of water in the reservoirs during the study.

**Table 2:** Summary of water treatment used in the 15 communities.

Community	Aeration	Settling pond	CuSO_4_	KMnO_4_	Activated carbon	Lime/soda ash	CO_2_	Flocculation (alum) settling	Sand filtration	Membrane filtration	Chlorination	Fluoridation	NH_3_
Manitoba													
1						X	X	X	X		X	X	
2				X	X	X	X	X	X		X	X	
3	X			X	X	X	X	X	X		X	X	
4	X				X				X		X		
Saskatchewan													
5	X		X		X			X	X		X		
6	X							X	X		X		
7		X	X		X			X	X		X		
8	X	X	X	X	X			X	X		X		
9				X	X			X	X		X		
10	X	X	X	X				X	X		X		
Alberta													
11				X	X			X			X	X	X
12	X	X			X			X		X	X	X	
13	X			X	X			X	X		X		
14	X	X			X				X		X		
15				X	X			X	X		X	X	X

Abbreviations: CO_2_, carbon dioxide; CuSO_4_, copper sulfate; KMnO_4_, potassium permanganate; NH_3,_ ammonia.

**Table 3:** Pesticides and degradation products monitored in reservoir water samples (*n* = 206).

Herbicide	Percent of samples with detection	Detection limit (ng/L)	No. of samples with detection	Maximum concentration (ng/L)
2,4-D	100	0.47	206	1,850
MCPA	99	0.58	205	374
Clopyralid	99	0.59	205	1,050
Dicamba	86	0.73	179	1,040
Diclorprop	82	0.42	171	113
Mecoprop	77	0.50	160	83.1
Bromoxynil	54	0.99	112	384
Ethametsulfuron	35	0.01	73	80.4
Atrazine	27	5.76	48	52.7
Tribenuron	20	0.01	42	30.1
Desethylatrazine	21	26.80	37	(20.8)^a^
Picloram	13	0.66	27	457
Imazamethabenz A	13	0.14	27	194
Desethylsimazine	12	148.00	22	(25.3)
2,4,5-T	11	0.39	23	4.18
Sulfosulfuron	10	1.0	21	36.1
Fenoprop	9	0.40	19	5.8
Imazamethabenz B	7	0.09	15	93.5
2,3,6-TBA	6	1.10	12	2.43
Imazethapyr	6	1.20	12	11.0
Thifensulfuron	3	1.0	7	12.0
Butylate	3	55.40	5	(3.12)
Metsulfuron	2	1.0	5	2.1
MCPB	2	0.63	5	12.8
Diclofop	2	42.30	4	(4.4)
Benzoylprop	1	26.20	2	(1.3)
Simazine	1	16.40	2	(13.8)
Triallate	1	4.14	2	(3.9)
Trifluralin	1	5.15	2	(1.0)
Metribuzin	< 1	20.7	1	185
Chlorpyrifos	3^b^	14.80	5	20.1
Dimethoate	1^b^	25.10	1	(5.98)

a Values in parentheses are estimates of concentration below the reporting limit.

b *n* = 30.

**Table 4:** Mean total pesticide concentrations (ng/L) and mean individual pesticide concentrations (± SD; ng/L) in 15 reservoirs in the three provinces (*n* = 163; sample from May 2003 to April 2004).

	Manitoba				Saskatchewan						Alberta				
Herbicide	1	2	3	4	5	6	7	8	9	10	11	12	13	14	15
Mean total concentration	302	341	274	199	98	316	172	498	176	128	271	125	1062	48	98
2,4-D	88 ± 42	131 ± 47	182 ± 107	46 ± 50	27 ± 12	254 ± 142	96 ± 35	121 ± 32	131 ± 28	28 ± 12	37 ± 28	83 ± 109	597 ± 199	17 ± 6.3	12 ± 6.0
MCPA	80 ± 39	32 ± 15	47 ± 36	49 ± 41	38 ± 20	15 ± 9.2	21 ± 9.7	263 ± 72	15 ± 3.6	43 ± 24	22 ± 29	20 ± 21	178 ± 52	16 ± 6.3	15 ± 12
Clopyralid	53 ± 27	64 ± 49	14 ± 4.9	35 ± 26	9.6 ± 8.3	3.6 ± 1.4	5.0 ± 1.6	91 ± 23	2.3 ± 1.0	35 ± 14	3.6 ± 1.6	7.1 ± 5.0	58 ± 19	7.0 ± 2.5	29 ± 10
Diclorprop	7.5 ± 3.5	64 ± 27	7.8 ± 4.6	19 ± 17	11 ± 4.6	23 ± 12	26 ± 10	5.6 ± 2.2	5.2 ± 1.0	1.4 ± 1.1	+	3.9 ± 3.1	+	+	+
Dicamba	16 ± 7.4	15 ± 5.4	5.4 ± 3.2	7.5 ± 8.0	1.8 ± 1.1	6.6 ± 6.6	9.3 ± 5.3	1.9 ± 1.3	14 ± 3.2	1.8 ± 1.5	2.1 ± 0.9	ND	1.1 ± 0.9	2.9 ± 0.7	+
Mecoprop	4.6 ± 3.6	1.4 ± 0.7	8.2 ± 7.5	3.0 ± 2.5	1.0 ± 0.8	3.8 ± 1.7	8.4 ± 6.1	4.9 ± 2.1	2.8 ± 1.1	11 ± 4.9	5.1 ± 5.2	1.3 ± 0.8	2.4 ± 1.6	2.5 ± 1.1	5.7 ± 3.8
Bromoxynil	5.8 ± 4.2	2.0 ± 1.7	2.6 ± 2.6	4.1 ± 3.4	3.4 ± 5.0	2.1 ± 1.7	1.6 ± 1.4	1.8 ± 1.6	1.0 ± 0.6	2.0 ± 2.5	2.1 ± 2.2	+	0.8 ± 0.5	+	ND
Ethametsulfuron	2.6 ± 1.5	6.3 ± 7.1	2.0 ± 3.2	2.6 ± 1.5	ND	ND	ND	4.9 ± 2.7	ND	2.1 ± 2.4	ND	ND	ND	ND	ND
Sulfosulfuron	1.4 ± 0.9	ND	ND	25 ± 8.6	ND	ND	ND	ND	ND	ND	ND	ND	ND	ND	ND
Atrazine	34 ± 15	13 ± 4.2	4.7 ± 2.1	+	+	+	+	+	+	ND	ND	+	ND	ND	ND
Tribenuron	0.5 ± 0.3	0.3 ± 0.7	ND	0.4 ± 0.3	ND	4.0 ± 1.0	ND	+	ND	+	ND	ND	ND	ND	ND
Picloram	ND	ND	ND	ND	ND	ND	ND	ND	ND	ND	167 ± 114	ND	216 ± 145	ND	+
2,3,6-TBA	ND	+	+	ND	ND	+	+	+	ND	+	+	ND	+	+	ND
2,4,5-T	ND	+	ND	+	+	+	ND	+	+	+	+	ND	1.3 ± 1.3	+	+
Imazamethabenz A	+	+	+	+	ND	ND	ND	+	ND	ND	ND	ND	+	ND	+
Imazamethabenz B	+	+	ND	+	ND	ND	ND	+	ND	ND	ND	ND	+	ND	ND
Imazethapyr	ND	ND	ND	ND	ND	+	ND	+	+	+	ND	ND	+	ND	ND
MCPB	ND	+	ND	ND	ND	ND	+	ND	ND	+	ND	ND	+	+	ND
Fenoprop	ND	+	ND	+	+	+	+	+	+	+	+	ND	0.9 ± 1.7	+	ND
Benzoylprop	ND	ND	ND	ND	ND	ND	ND	ND	ND	+	+	ND	ND	ND	
Butylate	ND	ND	+	ND	ND	ND	+	ND	+	ND	ND	ND	+	ND	ND
Desethylatrazine	+	+	+	+	+	+	+	+	+	+	+	+	+	+	+
Desethylsimazine	+	+	+	+	+	+	ND	ND	+	+	+	+	+	+	+
Diclofop	ND	ND	ND	ND	ND	ND	ND	ND	ND	ND	+	+	+	ND	+
Simazine	ND	ND	ND	ND	ND	ND	ND	ND	ND	+	ND	+	ND	ND	ND
Triallate	ND	ND	ND	ND	ND	ND	ND	ND	ND	ND		+	ND	+	ND
Trifluralin	+	ND	ND	ND	ND	ND	ND	ND	ND	ND	ND	ND	ND	ND	ND
Chlorpyrifos	ND	ND	ND	ND	ND	+	+	ND	+	ND	+	ND	ND	ND	ND
Dimethoate	ND	+	ND	ND	ND	ND	ND	ND	ND	ND	ND	ND	ND	ND	ND

Abbreviations: +, detections in < 50% of samples; ND, not detected.

**Table 5:** Mean herbicide concentrations in reservoirs, calculated herbicide reduction in water treatment facilities, herbicide concentrations in drinking water, and Canadian drinking water guideline values.

	Reservoirs	Water treatment plant			Drinking water			
Herbicide	Mean conc^a^ (ng/L; *n* = 163)	Mean percent reduction	Variability (range, %)	No. of paired samples	Calculated mean annual conc^b^ (ng/L; *n* = 163)	Calculated mean maximum conc^c^ (ng/L; *n* = 111)	Maximum conc^d^ in drinking water in July (ng/L; *n* = 28)	Guideline^e^ (ng/L)
2,4-D	123	39	0–84	28	75	364	589	100,000
MCPA	57	45	0–93	26	31	98	865	2,000^f^
Clopyralid	28	14	0–88	27	24	50	393	None
Dichlorprop	16	29	0–55	19	11	+	105	100,000^f^
Dicamba	6.6	38	0–95	19	4	+	748	120,000
Mecoprop	4.4	34	0–80	11	3	1.6	42	10,000^f^
Bromoxynil	2.4	46	0–98	12	1	+	227	5,000
Picloram	–	33	16–45	3	–	145	174	None
Imazethapyr	–	38	0–79	3	–	ND	3	None
Imazamethabenz A and B	–	77	65–93	3	–	+	101	None
Atrazine	–	44	0–71	5	–	ND	7.4	5,000
Ethametsulfuron	–	60	23–92	7	–	ND	4	None
Tribenuron	–	28	0–91	7	–	ND	4	None
Thifensulfuron	–	–	–	2	–	ND	< 2	None
Sulfosulfuron	–	86	100	1	–	ND	2.9	None
Metsulfuron	–	–	–	1	–	ND	< 2	None
2,3,6-TBA	–	–	–	–	–	+	3.8	None

Abbreviations: +, present at < 1 ng/L; –, insufficient data to calculate value for cell; conc, concentration; ND, not detected.

a Calculated from data in Table 4.

b Mean reservoir concentration adjusted for percent reduction.

c Concentrations for reservoir 13 (Table 4) adjusted for percent reduction.

d Maximum concentrations of individual herbicides in drinking water samples (*n* = 28).

e Data from CCME (1999).

f Data from World Health Organization (2004).

**Table 6:** Herbicide concentrations (ng/L) in April/May and July reservoir water samples, number of paired comparisons, and statistical *p*-values.

Pesticide	April/May (mean ± SE)	July (mean ± SE)	No. of paired samples	*p*-Value
Significantly greater in July				
Bromoxynil	1.5 ± 0.3	29.6 ± 12.5	36	< 0.001
MCPA	36.5 ± 8.7	89.1 ± 13.8	42	< 0.001
Diclorprop	9.1 ± 2.9	16.7 ± 3.1	40	< 0.001
Dicamba	11.3 ± 4.9	42.9 ± 26.0	39	< 0.001
2,4-D	78.9 ± 20.2	147.2 ± 43.9	42	< 0.001
Clopyralid	26.1 ± 6.3	55.8 ± 24.8	42	0.001
Not significant				
Mecoprop	6.1 ± 1.3	8.4 ± 2.3	42	0.08
Tribenuron	1.3 ± 0.2	4.9 ± 2.5	12	0.18
Ethametsulfuron	6.9 ± 1.9	8.5 ± 4.4	18	0.88
Imazamethabenz A	6.4 ± 1.7	22.2 ± 13.6	15	0.98

**Table 7:** Comparison of the total herbicide concentration (ng/L) in reservoir water and in drinking water from four reservoirs in Manitoba on 5 or 6 July after a dry (2004) or wet (2005) period.

	2004^a^		2005^a^	
Community	Total reservoir concentration	Drinking water concentration (no. of herbicides)	Total reservoir concentration	Drinking water concentration (no. of herbicides)
1	825	444 (8)	1,746	2,423^b^ (15)
2	347	177 (7)	1,733	578 (7)
3	213	184 (7)	2,269	1,532 (10)
4	395	145 (8)	876	161 (9)
Mean		(7.5)		(10.3)

Precipitation was measured by meteorologic stations 15 days before sample collection: 22.3 mm for 2004 and 133.3 mm for 2005.

a Number of drinking water samples = 4.

b Drinking water concentration > reservoir water concentration.
